# Genome-wide identification of the 14–3-3 gene family and its participation in floral transition by interacting with TFL1/FT in apple

**DOI:** 10.1186/s12864-020-07330-2

**Published:** 2021-01-08

**Authors:** Xiya Zuo, Shixiang Wang, Wen Xiang, Huiru Yang, Muhammad Mobeen Tahir, Shangong Zheng, Na An, Mingyu Han, Caiping Zhao, Dong Zhang

**Affiliations:** 1 College of Horticulture, Northwest A & F University, Yangling, 712100 China; 2 College of Life Sciences, Northwest A & F University, Yangling, 712100 China

**Keywords:** Apple, 14–3-3 s, Genome-wide, TFL1, FT, Floral transition

## Abstract

**Background:**

Apple (*Malus domestica* Borkh.) is a popular cultivated fruit crop with high economic value in China. Apple floral transition is an important process but liable to be affected by various environmental factors. The 14–3-3 proteins are involved in regulating diverse biological processes in plants, and some 14–3-3 members play vital roles in flowering. However, little information was available about the 14–3-3 members in apple.

**Results:**

In the current study, we identified eighteen 14–3-3 gene family members from the apple genome database, designated *MdGF14a* to *MdGF14r*. The isoforms possess a conserved core region comprising nine antiparallel α-helices and divergent N and C termini. According to their structural and phylogenetic features, Md14–3-3 proteins could be classified into two major evolutionary branches, the epsilon (ɛ) group and the non-epsilon (non-ɛ) group. Moreover, expression profiles derived from transcriptome data and quantitative real-time reverse transcription PCR analysis showed diverse expression patterns of Md14–3-3 genes in various tissues and in response to different sugars and hormone treatments during the floral transition phase. Four Md14–3-3 isoforms (*MdGF14a*, *MdGF14d*, *MdGF14i*, and *MdGF14j*) exhibiting prominent transcriptional responses to sugars and hormones were selected for further investigation. Furthermore, yeast two-hybrid and bimolecular fluorescence complementation experiments showed that the four Md14–3-3 proteins interact with key floral integrators, MdTFL1 (TERMINAL FLOWER1) and MdFT (FLOWERING LOCUS T). Subcellular localization of four selected Md14–3-3 proteins demonstrated their localization in both the cytoplasm and nucleus.

**Conclusion:**

We identified the Md14–3-3 s family in apple comprehensively. Certain Md14–3-3 genes are expressed predominantly during the apple floral transition stage, and may participate in the regulation of flowering through association with flower control genes. Our results provide a preliminary framework for further investigation into the roles of Md14–3-3 s in floral transition.

**Supplementary Information:**

The online version contains supplementary material available at 10.1186/s12864-020-07330-2.

## Background

The 14–3-3 family, consisting of multiple subunits, is present in all eukaryotic organisms, such as yeast [1], humans [2], and *Arabidopsis* [3]. Initially, they were identified as essential components of the protein/G box complex in *Arabidopsis* and were thus named as “G box factor 14-3-3,” or “GF14” [4]. 14–3-3 proteins belong to a highly conserved protein family and regulate multiple cellular processes through interactions with other proteins. In plants, 14–3-3 s usually exist in the form of homodimers or heterodimers [5]; each subunit is able to bind a separate phosphorylated target protein by the recognized binding motifs, namely mode I (RXX (pS/pT) XP or modified motif LX(R/K) SX (pS/pT)XP), and mode II (RX(F/Y) X (pS)XP) [6, 7]. More recently, a mode III (SW (pT)X-COOH) motif has also been defined [8]. However, a few 14–3-3 binding proteins do not match these phosphorylated consensus motifs, and in some cases the binding does not depend on phosphorylation of the target proteins [9].

In general, 14–3-3 proteins serve as molecular escorts and regulate the function of targets through physical obstruction, scaffolding, or distorted conformational changes. The effects caused by 14–3-3 proteins can alter the stability, enzymatic activity, and subcellular localization of their binding partners, allowing them to respond quickly and accurately to altered signals [8, 10, 11]. For instance, in *Arabidopsis*, the activity of the plasma membrane H + -ATPase is mediated positively through direct associations with 14–3-3 proteins [10]. Additionally, a recent study reported that the phosphorylated transcription factor PHYTOCHROME-INTERACTING FACTOR 7 (PIF7) can be sequestered in the cytoplasm by 14–3-3 proteins [12].

During growth and development, plants sense environment signals constantly, leading to changes to biological processes in vivo, such as signaling pathways and metabolic regulation. In particular, plant 14–3-3 s are important regulators of membrane transport and nutrition metabolism. For example, 14–3-3 directly or indirectly interacts with proteins involved in nitrogen and carbon metabolism, thereby affecting plant nutrient metabolism pathways [13]. In plants, 14–3-3 complexes are involved in cell signals, stress responses, and transcriptional regulation [11, 14–17]. Several studies also established a role for 14–3-3 proteins in hormonal signaling, such as gibberellins (GA), brassinosteroids (BR), abscisic acid (ABA), cytokinins, and auxin [18]. In tobacco, 14–3-3 proteins can control gibberellin levels by binding to a bZIP transcriptional activator RSG (REPRESSION OF SHOOT GROWTH) [8, 19]. Interactions between 14 and 3-3 proteins and members of the ABA responsive-element binding factor (ABF) family are involved in the regulation of GA and ABA signaling [20]. Moreover, 14–3-3 proteins can alter the localization and activity of the transcription factor BRASSINAZOLE RESISTANT 1 (BZR1) in the BR signaling pathway [21]. All of the above studies demonstrated the key role of 14–3-3 proteins in the cross-talk among these pathways.

Recent studies indicated that 14–3-3 proteins can affect the transition from vegetative growth to reproductive growth, which is strictly controlled by both environmental and endogenous conditions. In *Arabidopsis*, research into photoperiodic flowering control indicated that 14–3-3 *ν* and *μ* proteins interact with CONSTANS (CO) [22], a major regulator of the photoperiodic pathway, that directly activating *FT* expression for flowering. Mutant plants with T-DNA insertions for 14–3-3 *ν* and *μ* showed slightly late flowering [22]. In rice, compared with wild-type plants, transgenic plants overexpressing GF14c (a rice 14–3-3 protein) exhibited delayed flowering, while the knockout mutants displayed early flowering [11]. Moreover, in plants, 14–3-3 proteins have been reported to physically interact with floral integrators, FLOWERING LOCUS T (FT) and TERMINAL FLOWER1(TFL1) [23, 24].

FT and TFL1, which belong to the same phosphatidylethanolamine binding protein (PEBP) family, share similar amino acid sequences; however, they have antagonistic roles in flower induction. FT over-expression in apple leads to early flowering [25]. FT is mainly expressed in leaves, and its protein is transported to the shoot apical meristem (SAM) for a long distance in phloem to induce flowering [26–28]. Loss of TFL1 function results in an early-flowering phenotype and severely shortened juvenile period in apple [29, 30]. The ectopic expression of apple TFL1 in *Arabidopsis* showed obvious late flowering phenotype [31]. *TFL1* mRNA is weakly expressed in the center of the SAM during the vegetative phase and is strongly upregulated at floral transition, thereby inducing the regulation of flowering time [31, 32]. In previous reports, TFL1 and FT interact with the bZIP transcription factor FD [33, 34], which regulates the transcription of the floral identity gene, *APETALA1 (AP1)*, leading to flowering [33, 35]. FT forms a florigen activation complex together with 14–3-3 proteins and FD. In contrast, TFL1 forms a florigen repression complex to repress FT [35–37]. Hence, the interactions of TFL1/FT with FD are mediated by 14–3-3 proteins.

Apple is a widely cultivated profitable fruit tree worldwide. Apple floral transition is a serious problem and some apple varieties, such as Fuji, are susceptible to alternate bearing, which directly causes production fluctuations. The flowering process is markedly affected by nutrient conditions and hormonal signals [38–40]. 14–3-3 proteins are known to influence flowering by integrating multiple signals [22, 35, 36]. Studies have revealed many details of the functions of 14–3-3 proteins in *Arabidopsis* [5], Rice [41], Soybean [42], Tomato [43], and *Populus* [44]. However, the diversity of 14–3-3 proteins in apple, and their potential roles in apple floral transition, remain unknown. In the present study, we identified 18 apple 14–3-3 genes and analyzed their chromosomal locations, gene structures, and evolutionary relationships in detail. Global expression profiles were determined to assess their responses to treatment with sugars and plant hormones. Their subcellular localizations in tobacco leaves were also detected. Furthermore, we confirmed MdTFL1 (MdTFL1–1 and MdTFL1–2) and MdFT as 14–3-3 s binding partners using yeast two-hybrid and bimolecular fluorescence complementation (BiFC) assays. Identification of apple 14–3-3 family members and their interactions with target proteins laid the foundation for further understanding of the 14–3-3 gene family in apple.

## Results

### Genome-wide identification and chromosomal locations of 14–3-3 genes in the apple genome

To identify 14–3-3 family members in apple, previously published 15 *Arabidopsis* 14–3-3 protein sequences were used as queries against the Apple Genome Database using the BLASTp program (E-value <1e-5). After manually removing sequences containing an incomplete 14–3-3 domain, 18 putative Md14–3-3 genes were identified, which were named *MdGF14a–MdGF14r* based on their chromosomal positions (Table 1; Additional file 1: Figure S1). The 18 Md14–3-3 genes identified were located on 9 of the 17 chromosomes of apple, and 2 genes (*MdGF14a* and *MdGF14b*) were mapped on unanchored scaffolds. The basic information of these Md14–3-3 genes is provided in Table 1. The putative Md14–3-3 proteins contained 252 (MdGF14f and MdGF14h) to 302 (MdGF14q) amino acid residues.

**Table 1:** Information on Apple 14–3-3 genes

Name	Gene identifier	CDS (bp)	Peptide (aa)	Gene locationa	Strand
MdGF14a	MD00G1018600	786	261	3,143,713–3,146,570	–
MdGF14b	MD00G1176300	798	265	40,806,633–40,810,797	+
MdGF14c	MD01G1037700	762	253	13,038,612–13,047,589	–
MdGF14d	MD05G1301400	786	261	43,423,732–43,426,889	–
MdGF14e	MD07G1096000	789	262	10,468,901–10,472,640	+
MdGF14f	MD08G1184000	759	252	22,921,142–22,936,861	–
MdGF14g	MD08G1187400	795	264	23,593,019–23,594,916	+
MdGF14h	MD08G1193600	759	252	24,908,573–24,911,343	–
MdGF14i	MD10G1084500	786	261	12,408,819–12,411,711	+
MdGF14j	MD10G1280300	786	261	37,120,031–37,122,984	–
MdGF14k	MD13G1063600	792	263	4,396,805–4,399,529	–
MdGF14l	MD15G1315300	765	254	32,152,582–32,157,628	+
MdGF14m	MD15G1370500	768	255	45,033,182–45,036,653	–
MdGF14n	MD15G1373400	792	263	45,588,403–45,590,135	+
MdGF14o	MD16G1062100	849	282	4,419,295–4,422,061	–
MdGF14p	MD17G1074900	786	261	6,068,710–6,072,300	–
MdGF14q	MD17G1105100	909	302	8,914,153–8,917,345	+
MdGF14r	MD17G1231000	783	260	27,941,075–27,945,515	+

### Gene structure and multiple sequence alignment of 14–3-3 genes

**Fig. 1:**
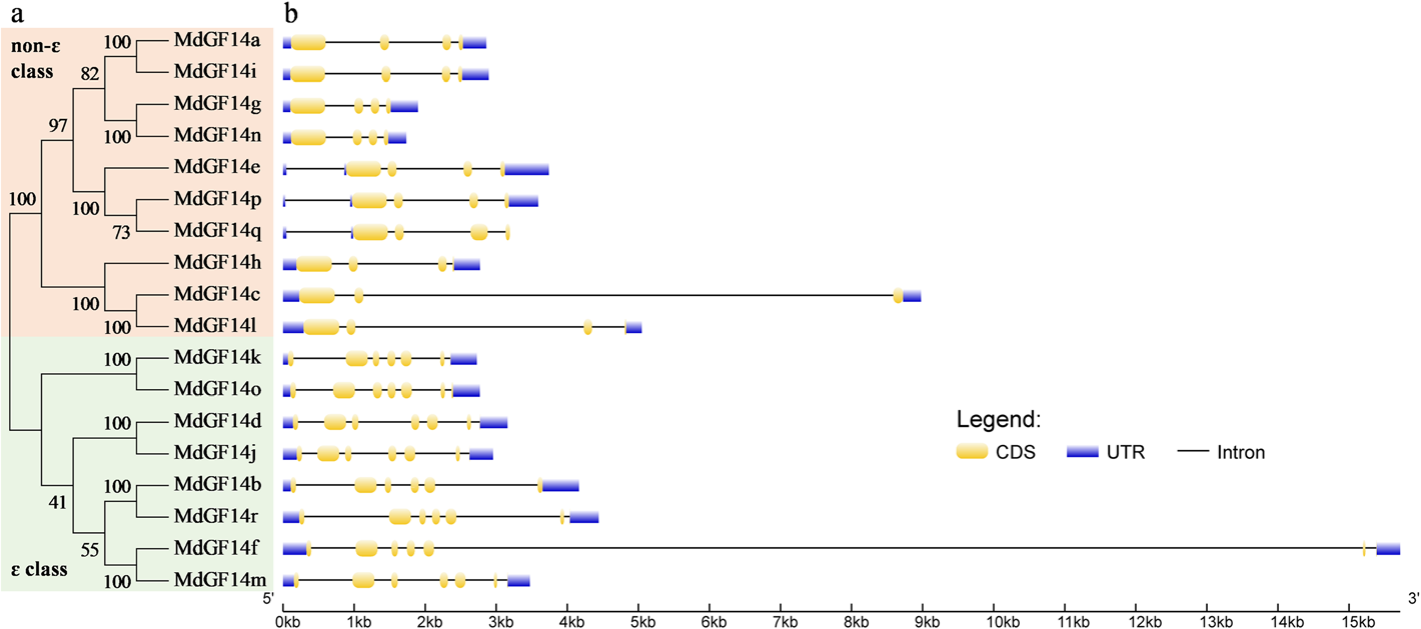
Analysis of Md14–3-3 gene structures. **a** The unrooted phylogenetic tree of Md14–3-3 protein sequences was constructed using the maximum likelihood method in MEGA 7.0 software. The two major groups are marked with pink or light green. **b** Exon-intron composition of Md14–3-3 genes. The blue and yellow boxes, and black lines, represent UTR, exon, and intron positions, respectively

To determine the gene structures of Md14–3-3 family members, we investigated the divergence of Md14–3-3 s exon-intron structures (Fig. 1), revealing the evolutionary relationships. The full-length amino acid sequences of Md14–3-3 proteins were used to construct the phylogenetic tree using the maximum likelihood method in the MEGA7.0 software. As shown in Fig. 1a, the Md14–3-3 family members grouped into two major evolutionary branches, the ɛ group and the non-ɛ group. The ɛ group comprised the isoforms *MdGF14k*, *MdGF14o*, *MdGF14d*, *MdGF14j*, *MdGF14b*, *MdGF14r*, *MdGF14f*, and *MdGF14m*. The non-ɛ group comprised the isoforms *MdGF14a*, *MdGF14i*, *MdGF14g*, *MdGF14n*, *MdGF14e*, *MdGF14p*, *MdGF14q*, *MdGF14h*, *MdGF14c*, and *MdGF14l* (Fig. 1a). Moreover, the ɛ group was separated into four well-supported subbranches. The non-ɛ group was also separated into four very distinct subbranches. The ɛ and non-ɛ groups are well supported by the intron-exon structure. The ɛ members have six exons and six introns (including an additional 3′ intron). In contrast to the ɛ group, most non-ɛ members contain four exons and three introns, except for *MdGF14c* with three exons, and *MdGF14e*, *MdGF14p*, and *MdGF14q* containing an extra intron in the 5′ leader (Fig. 1b). To detect the sequence conservation of 14–3-3 family members, we performed multiple sequence alignment of the 18 full-length Md14–3-3 protein sequences (Additional file 2: Figure S2). Notably, the amino acid sequences of the N-terminal and C-terminal regions are significantly different, with little amino acid conservation, while the central regions comprise nine antiparallel α-helices (α1-α9) and are relatively conserved (Additional file 2: Figure S2), especially the α1, α3, α5, α7, and α9 domains, whose functions might have been conserved during evolution.

### Phylogenetic and synteny analysis

**Fig. 2:**
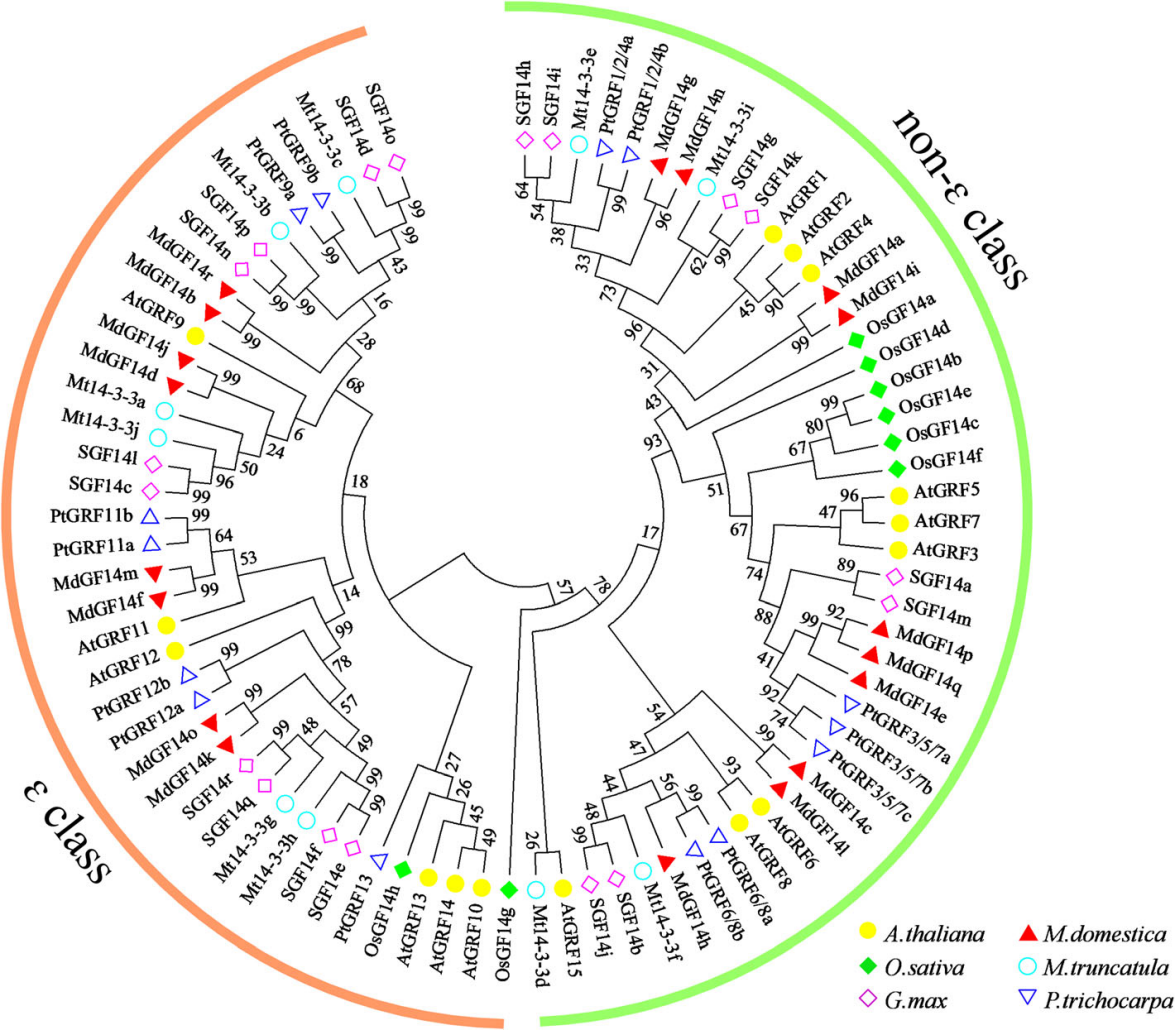
Phylogenetic tree showing the evolutionary relationships of Md14–3-3 proteins in apple and other plant species. The maximum likelihood method was used to build the phylogenetic tree using the MEGA7.0 program. Protein designations consist of the prefixes *A. thaliana,* (At, yellow circles), *M. domestica,* (Md, red triangles), *O. sativa,* (Os, green rhombus), *M. truncatula,* (Mt, blue circles), *G. max* (Gm, pink rhombus) and *P. trichocarpa* (Pt, blue triangles). Detailed information for 14–3-3 s from these plant species are listed in Additional file 3: Table S1

To gain further insights into the evolutionary relationships of 14–3-3 proteins in different species, we constructed a phylogenetic tree by maximum likelihood method using the 14–3-3 protein sequences from six plant species: *Arabidopsis thaliana*, *Malus domestica*, *Oryza sativa*, *Medicago trucatula*, *Glycine max*, and *Populus trichocarpa* (Fig. 2). The detailed information of all 14–3-3 genes identified in this study is provided in Additional file 3: Table S1. As shown in the phylogenetic analysis (Fig. 2), the 14–3-3 family members from the six plant species were divided into two major classes (ɛ class and non-ɛ class), as described previously [3].

**Fig. 3:**
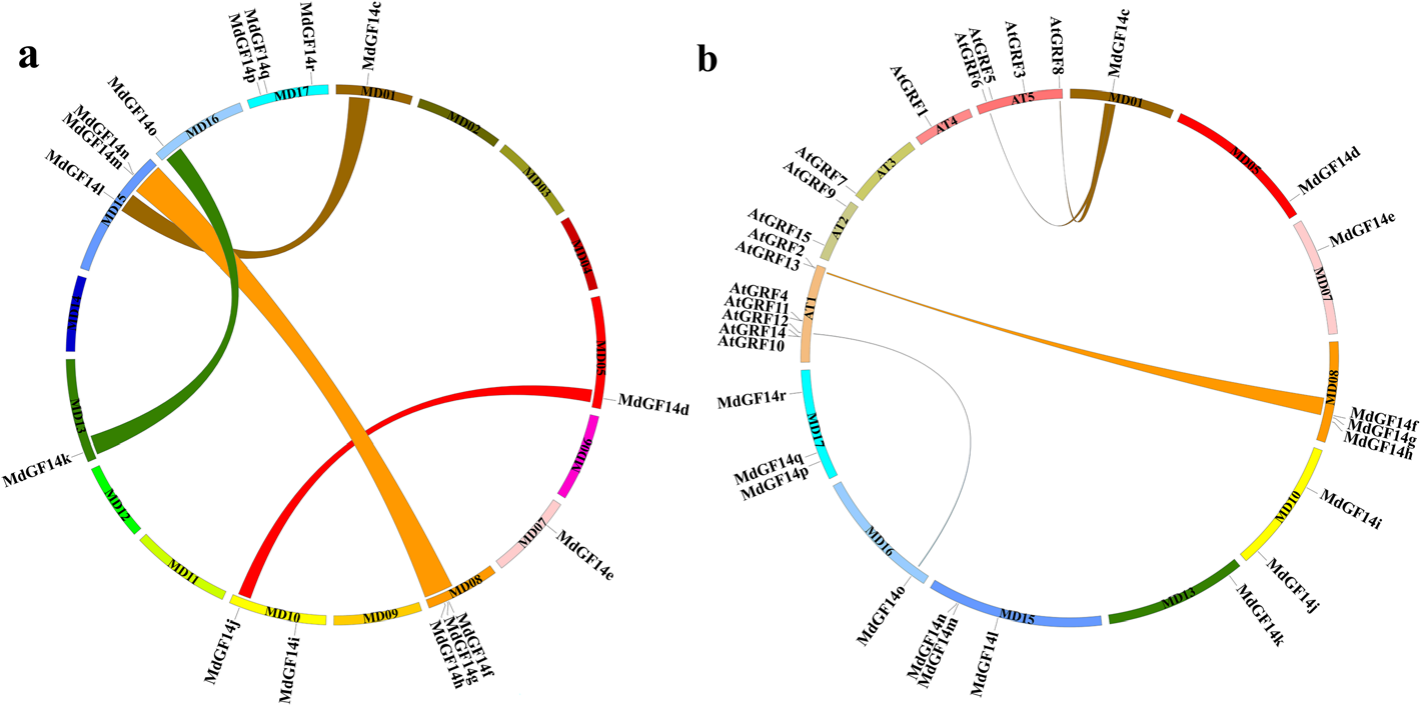
Syntenic relationships of apple and *Arabidopsis* 14–3-3 genes. **a** Chromosomal distribution and duplication relationships of Md14–3-3 genes. **b** Syntenic relationship between apple and *Arabidopsis*. The colored curves represent apple and *Arabidopsis* syntenic gene regions. The graph was generated using Circos (version 0.63)

The evolution and expansion of gene families are closely related to the occurrence of tandem duplication and segmental duplication events. Tandem duplication is usually characterized by multiple members of a family forming gene clusters in the same intergenic region. Segmental duplication, which occurs most frequently in plants, might cause scattered family members on different chromosomes [45]. To understand the expansion patterns of the Md14–3-3 genes in the apple genome, we analyzed tandem and segmental duplications. As shown in Fig. 3a, four Md14–3-3 genes (*MdGF14m*/*MdGF14n* and *MdGF14g*/*MdGF14f*) were clustered into two tandem duplication regions on linkage groups 08 and 15 in apple. In addition, the *MdGF14l/MdGF14c*, *MdGF14k/MdGF14o*, and *MdGF14j/MdGF14d* gene pairs might have been generated by segmental duplications, because they are located on different and non-homologous chromosomes (Fig. 3a). Additionally, a syntenic map of 14–3-3 genes in apple and *Arabidopsis* was created. A total of four pairs of orthologous genes (*MdGF14o-AtGRF12*, *MdGF14c-AtGRF6/AtGRF8*, *MdGF14f-AtGRF13*, *and MdGF14g-AtGRF2*) were identified (Fig. 3b). These results indicated that some Md14–3-3 genes were possibly generated by gene duplication, which is a major driving force for Md14–3-3 evolution. Thus, synteny analysis and phylogenetic comparison of Md14–3-3 genes provided a deep insight into their evolutionary characteristics.

### *Cis*-elements in the promoters of Md14–3-3 genes

To further explore the function and regulatory patterns of Md14–3-3 genes, the intergenic regions at 2000 bp upstream from the start codon of the 18 Md14–3-3 genes were scanned for putative *cis*-regulatory elements using the PlantCARE database. A series of *cis*-acting elements involved in hormonal responses, and light and abiotic stress responses were found in the promoter regions of these Md14–3-3 genes (Additional file 4: Table S2). Among the *cis*-acting regulatory elements involved in hormone responses, abscisic acid responsive elements (ABREs) were present in almost all members of the Md14–3-3 family, except *MdGF14i*. In addition, the numbers of hormone-related *cis*-regulatory elements varied greatly among the Md14–3-3 genes. For example, four gibberellin response elements (P-box) were present in the *MdGF14r* promoter, but none were found in the promoters of *MdGF14g*, *MdGF14h*, *MdGF14k*, or *MdGF14n*. MeJA-related elements (CGTCA-motif and TGACG-motif), auxin-responsive elements (AuxRR-core and TGA-element), and salicylic acid-related elements (TCA-element) were also observed in the promoters of 14, 11, and 12 Md14–3-3 genes, respectively. Moreover, light-responsive *cis*-elements were the most abundant among all 14–3-3 genes, including G-boxes, Box 4, AE-boxes, TCCC-motifs, GATA-motifs, I-boxes, TCT-motifs, and AT1-motifs, which may reflect the response of the 14–3-3 genes to light signals to regulate plant growth. Circadian-responsive elements were identified in the upstream flanking regions of *MdGF14d*, *MdGF14m*, *MdGF14p*, and *MdGF14q*. Meanwhile, stress response (e.g., drought and low temperature) elements were identified in the promoter sequences of certain Md14–3-3 genes (Additional file 4: Table S2). The presence of abundant elements in the promoters suggested that the 14–3-3 genes encode proteins that are involved in multiple biological processes.

### Expression profiles of Md14–3-3 genes in RNA-seq datasets

**Fig. 4:**
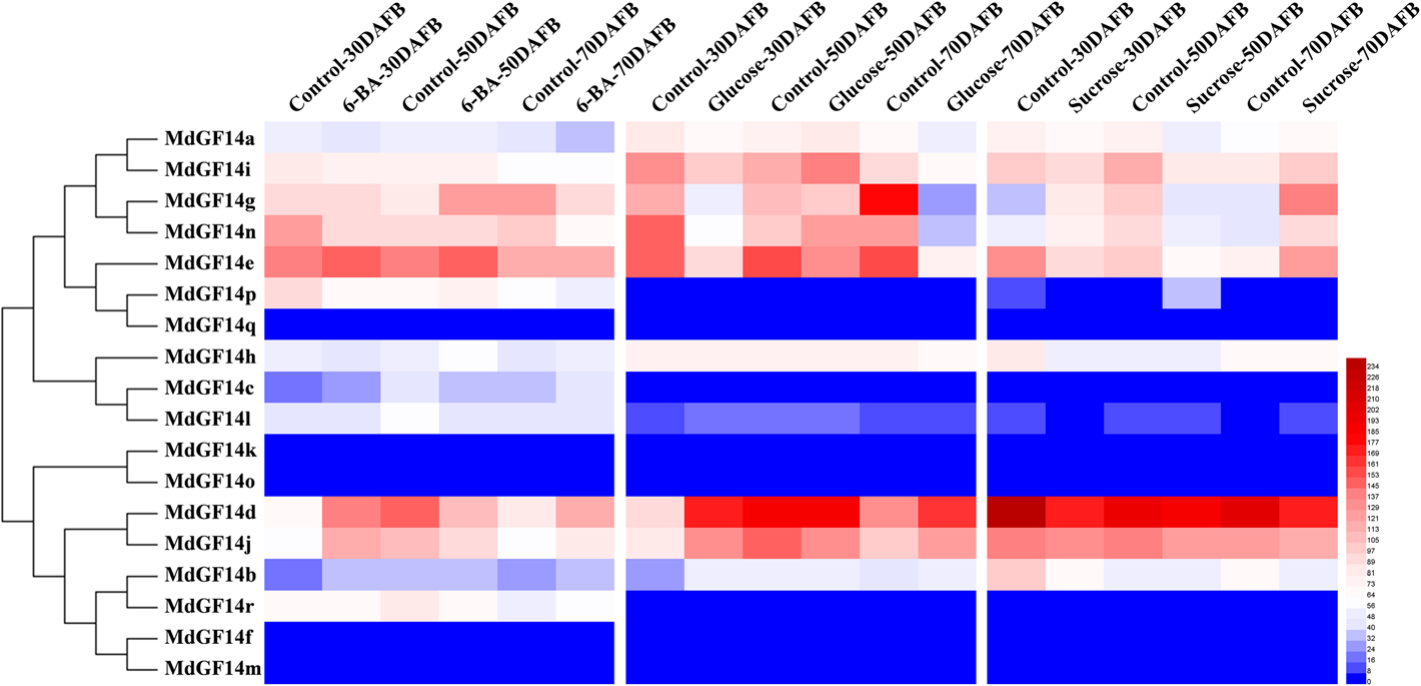
Expression profiles of Md14–3-3 s during the floral transition phase under 6-BA treatment, and glucose and sucrose treatment. For the 6-BA and glucose treatments, RNA-seq datasets for the expression profiles were retrieved from NCBI Sequence Read Archive (SRA) datasets (SRR6510620 [47] and SRP226830, respectively). For sucrose treatment (RNA-seq datasets not shared online), trees were sprayed twice at 29 and 36 days after full bloom (DAFB) with 15,000 and 20,000 mg L^− 1^ sucrose. Water was used as control. Samples of the terminal buds of the short shoots (< 5 cm) were collected at three time points (30, 50, and 70 DAFB) during floral transition. FPKM values were used to generate their expression profiles. The diagram was drawn using Heml 1.0 software. For other details, see Additional file 5: Table S3

Some reports claimed that 14–3-3 genes were involved in plant hormonal responses, such as to cytokinins, GA, and ABA [16, 18, 21] as well as sugar metabolism [43, 46]. To further determine the potential role of Md14–3-3 s genes in the context of apple flower induction, we performed a preliminary analysis of the expression profiles of the 18 Md14–3-3 genes in response to 6-benzylaminopurine (6-BA), glucose, and sucrose treatments, based on the transcriptomic sequence databases. For 6-BA and glucose treatment, RNA-seq datasets were retrieved from the NCBI Sequence Read Archive (SRA) datasets (SRR6510620 [47] and SRP226830, respectively). Glucose treatments (15,000 and 30,000 mg L^− 1^) were sprayed onto ‘Nagafu No. 2’ trees at 25 and 30 days after full bloom (DAFB), respectively. For sucrose treatment (RNA-seq datasets not shared online), 15,000 and 20,000 mg L^− 1^ sucrose was sprayed twice, at approximately 29 and 36 DAFB, respectively. Samples of the short shoot apex were collected at 30, 50, and 70 DAFB during floral transition. The fragments per kilobase of transcript sequence per million base pairs sequenced (FPKM) values of the Md14–3-3 genes are listed in Additional file 5: Table S3, and a heat map was generated to display their expression profiles (Fig. 4). In the non-ε group, the expression levels of *MdGF14a* and *MdGF14i* were down-regulated slightly, but not significantly, at the early stage of flower induction under these treatments. *MdGF14g* and *MdGF14n* exhibited similar expression patterns, both were differentially expressed at a certain processing time*. MdGF14e* was inhibited upon glucose and sucrose treatments, but there was no significant change after 6-BA treatment. In the ε group, the expression levels of *MdGF14d* were significantly higher, and were induced or inhibited by 6-BA and sugar at one or more time points, the same as its close paralog, *MdGF14j* (Fig. 4). This indicated that they may have similar functions. By contrast, genes in other subbranches of the ε class showed relatively low expression levels, especially *MdGF14o* (FPKM < 1), indicating that it might not function to a large extent in flower development. Overall, Md14–3-3 s showed different and multiple expression patterns in the transcriptome data, implying their functional diversity.

### Expression patterns of Md14–3-3 genes in various tissues and their responses to GA_3_ treatment as assessed using qRT-PCR

**Fig. 5:**
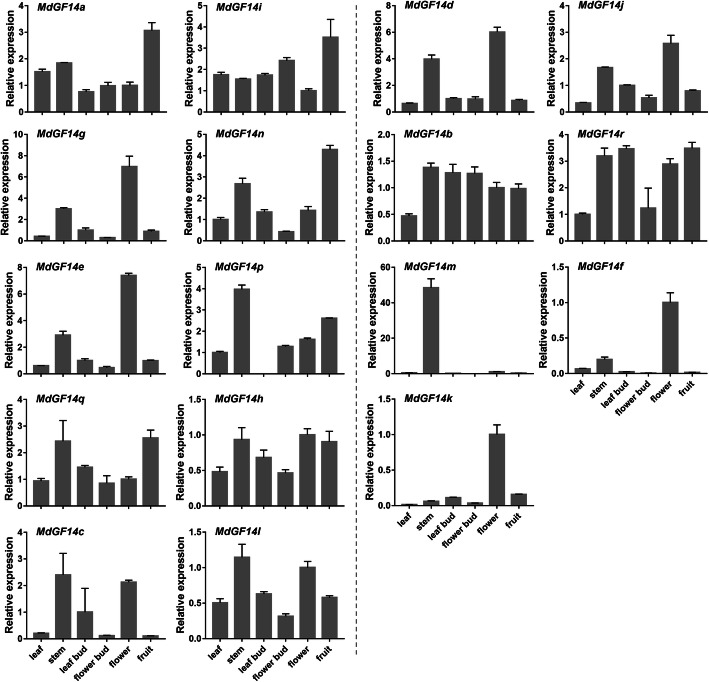
Quantitative real-time PCR analysis of Md14–3-3 gene expression in different tissues. Leaves were collected from the adjacent terminal buds. Stems were collected from new shoots grow in the spring. Leaf buds were collected from bourse shoots apices. Flower buds were also collected from full buds of spur apex. The developing fruit was collected after 40 DAFB. The expressions of Md14–3-3 members are arranged according to gene relationships (as shown on the left side of the dotted line, the non-ε group; on the right, the ε group). Each value represents the standard error of three replicates

To investigate the possible roles of the Md14–3-3 proteins, tissue-specific (leaves, stems, leaf buds, flower buds, flowers, and fruit) gene expression was determined using quantitative real-time reverse transcription PCR (qRT-PCR) (Fig. 5, Additional file 6: Table S4). As shown in Fig. 5, certain Md14–3-3 genes exhibited similar expression patterns in different tissues, while other Md14–3-3 s showed tissue-specific transcript accumulation patterns, suggesting the functional divergence of Md14–3-3 proteins. For example, genes with closer relationships (*MdGF14a* and *MdGF14i*) showed similar expression patterns, and both were expressed at higher levels in the tested tissues (Fig. 5), demonstrating that their encoded proteins might play similar roles in tissue development. Besides, two pairs of Md14–3-3 s in the segmental duplication group also showed similar expression patterns (Fig. 5). For example, *MdGF14d* and *MdGF14j* with similar gene structure were mainly expressed in stems and flowers. *MdGF14c* and *MdGF14l* showed relatively high expression levels in the stem. However, some genes in tandem duplicated regions displayed different expression patterns (Fig. 5). *MdGF14g* and *MdGF14n* displayed higher expression levels in flowers and fruit, respectively. *MdGF14m* was expressed at a very higher level in the stem compared with that in other tissues, while *MdGF14f* was highly expressed in flowers. Furthermore, two genes with a close relationship in the ε class, *MdGF14b* and *MdGF14r*, showed similar expression levels and were ubiquitously high expressed in nearly all tested tissues. Notably, transcription level of *MdGF14o* alone could not be detected in any of the selected tissues by qRT-PCR, suggesting its very low abundance. Similarly, in soybean, both *SGF14q* and *SGF14r*, the closest homologs of *MdGF14o*, were not detected in an expressed sequence tag (EST) database [42]. Some Md14–3-3 genes showed a very high expression level in specific tissues (Fig. 5). For example, *MdGF14k* exhibited strong preferential expression in flowers, signifying the putative role of its encoded protein in the regulation of flower development. These results indicated that some Md14–3-3 proteins play multiple important roles in apple growth and development.

**Fig. 6:**
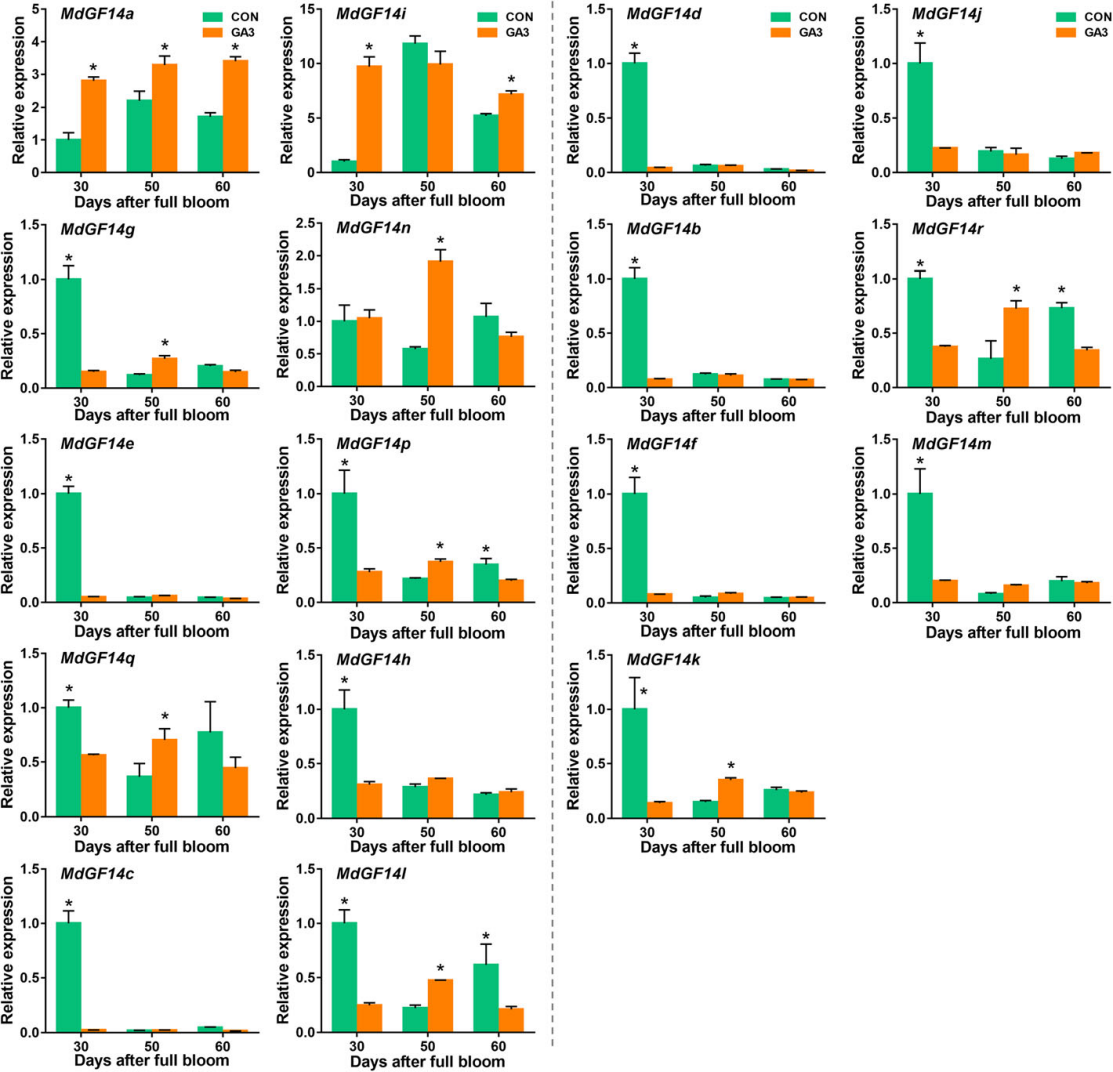
Quantitative real-time PCR analysis of Md14–3-3 gene expression in apple buds treated with gibberellin (GA_3_). GA_3_ at 500 mg L^− 1^ was sprayed on ‘Nagafu No. 2’ trees in a clear morning at 25 and 30 DAFB. At the same time, water was sprayed as a control. After treated twice, samples of the spur terminal buds were collected at 30, 50, and 70 days after full bloom (DAFB). Each value represents the standard error of three replicates. The expression profiles of genes were compared in the context of the gene relationships (the non-ε group on the left and the ε group on the right). The significance of the difference between the control and treatment groups was estimated by Student *t*-test at each date (*, *p*-value < 0.05)

In apple, gibberellin promotes vegetative growth, but inhibits floral transition, resulting a significant reduction in fruit load in the following year [40]. To assess the effect of exogenous GAs on gene expression, we applied GA_3_ (500 mg L^− 1^) spray treatment on the ‘Nagafu No. 2’ tree at approximately 25 and 30 DAFB. The spur terminal buds were collected at 30, 50, and 70 DAFB for further analysis. qRT-PCR was used to analyze the expression of the Md14–3-3 genes in response to GA_3_ (Fig. 6). In the non-ε class, significant upregulation of *MdGF14a* and *MdGF14i* was observed at 30 DAFB after GA_3_ treatment (Fig. 6). By contrast, the expression levels of several Md14–3-3 genes, including *MdGF14g*, *MdGF14e*, *MdGF14p*, *MdGF14h*, *MdGF14c*, and *MdGF14l* were markedly reduced and remained at a low level. The transcription level of *MdGF14n* did not differ significantly at first; however, subsequently, it increased by 4-fold at the second sampling point after GA_3_ treatment (Fig. 6). Interestingly, in the non-ε class, except for *MdGF14o*, which was not expressed at the various stages of flower bud development under GA_3_ treatment, all the other genes showed highly similar expression patterns during flower induction, displaying significant downregulation at 30 DAFB after treatment (Fig. 6), indicating that they might have similar roles in hormonal stress responses or apple development.

### Md14–3-3 s can interact with MdTFL1, and MdFT

**Fig. 7:**
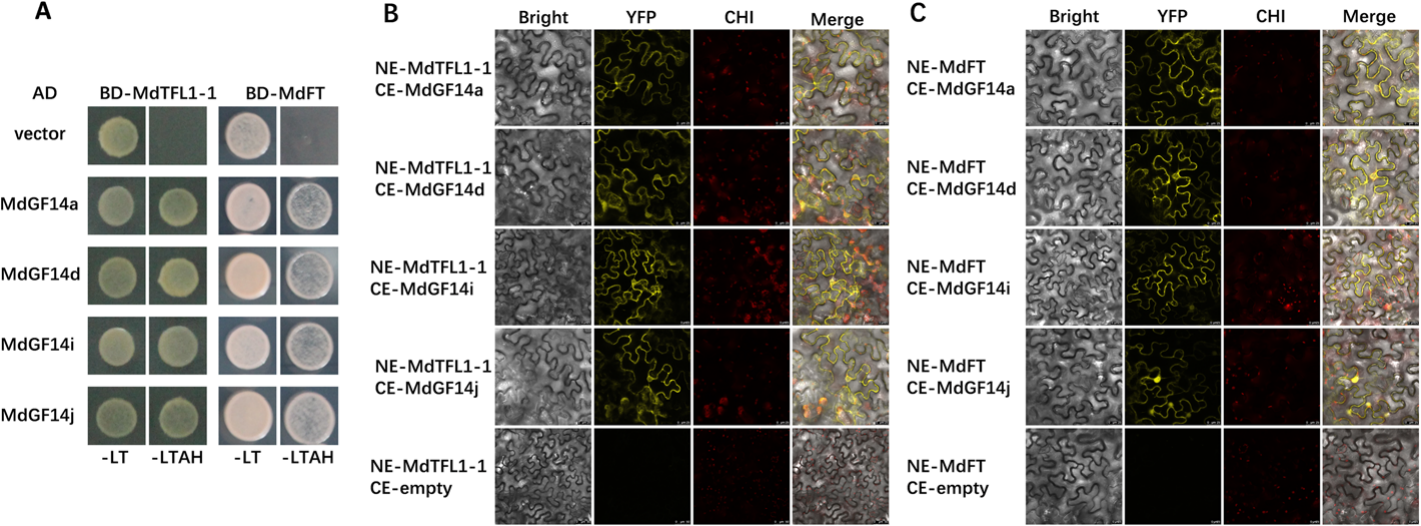
Yeast two-hybrid and BiFC assays of interactions between candidate Md14–3-3 proteins and MdTFL1/MdFT. **a** Yeast two-hybrid assays. MdTFL1–1, MdFT, and Md14–3-3 proteins were fused to the GAL4 DNA-binding domain (BD) or activation domain (AD) to generate the bait constructs or prey constructs. The empty pGADT7 vector was used as control. -LT, yeast medium lacking leucine and tryptophan. -LTAH, yeast medium lacking leucine, tryptophan, adenine and histidine. **b**, **c** BiFC assays in the *Nicotiana benthamiana* leaves. Interactions between Md14–3-3 s and MdTFL1–1 (**b**), and MdFT (**c**), respectively. MdTFL1–1 and MdFT coding regions were cloned into pSPYNE respectively, and MdGF14a, MdGF14d, MdGF14i, and MdGF14j coding regions were independently cloned into the pSPYCE vector. The empty pSPYCE vector served as the control. The negative control of empty pSPYNE vector was shown in Additional file 7: Figure S3. Fluorescence was imaged by laser scanning confocal microscopy (LEICA TCS SP8, Germany). The YFP fluorescence, chlorophyll autofluorescence (CHl), and bright-field images were merged. Bar = 25 μm

To address how Md14–3-3 s participate in floral transition, we focused on the floral pathway integrators, TFL1 and FT. Previously, we used the full-length MdTFL1 protein as a bait protein to conduct yeast two-hybrid screening in an apple flower bud cDNA library, and identified MdGF14a and MdGF14j. In addition, MdGF14i and MdGF14d are closely related to MdGF14a and MdGF14j, respectively (Fig. 1), and they exhibited prominent transcriptional responses to sugars and hormones. Therefore, we chose these four genes for further analysis. Previous studies showed interactions between 14 and 3-3 protein and TFL1or FT [11, 35, 36]. In apple, there exist two MdTFL1 encoding genes, *MdTFL1–1* and *MdTFL1–2* [48]. We repeated the yeast two-hybrid assay and further confirmed that both MdTFL1–1 and MdTFL1–2 proteins could interact with four 14–3-3 isoforms (MdGF14a, MdGF14d, MdGF14i, and MdGF14j, see Fig. 7a and Additional file 7: Figure S3). Moreover, the 14–3-3 isoforms preference for MdTFL1 was comparable to that of MdFT: The four 14–3-3 isoforms also interacted with MdFT in the yeast two-hybrid assays (Fig. 7a).

In addition, we used a BiFC assay to determine the interactions between Md14–3-3 proteins and MdTFL1 or MdFT in *Nicotiana benthamiana* leaves (Fig. 7, Additional file 7: Figure S3). The fluorescence signals from MdTFL1–1-Md14–3-3 s, MdTFL1–2-Md14–3-3 s, and MdFT-Md14–3-3 s interactions were detected in the cytoplasm and the nucleus, but mainly in the cytoplasm. Thus, these results clearly showed that Md14–3-3 proteins can interact with MdTFL1 and MdFT in yeast and plant cells.

### Subcellular localization of 14–3-3 proteins

**Fig. 8:**
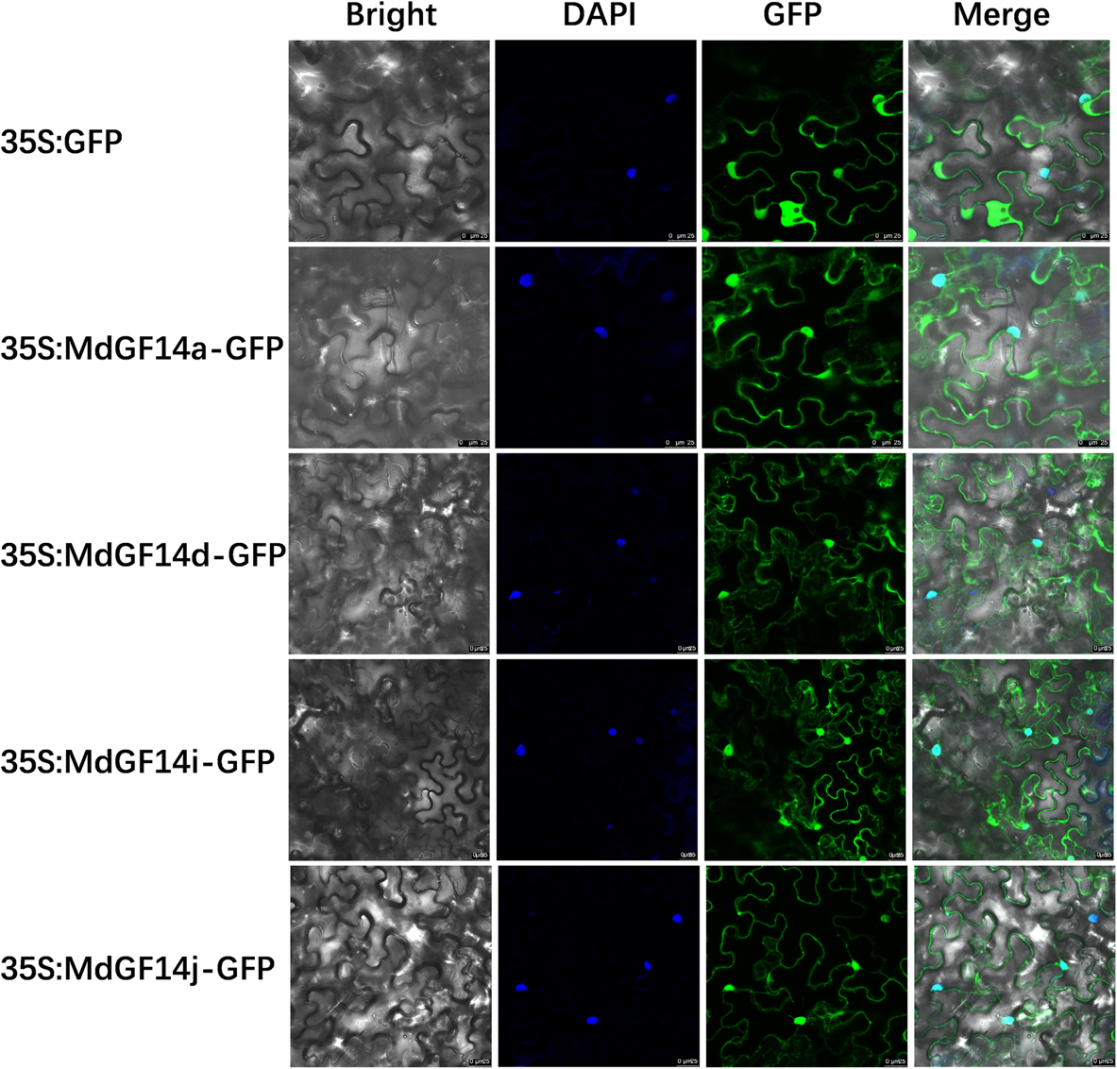
Subcellular localization of the four Md14–3-3 s proteins (MdGF14a, MdGF14d, MdGF14i, and MdGF14j) in *Nicotiana Benthamiana* leaves. All candidate genes were independently cloned into vector pCAMBIA2300 in which they were fused with green fluorescent protein (GFP) coding region. Free GFP was used as the control. DAPI (4′,6-diamidino-2-phenylindole) was used for the nucleus staining. The GFP fluorescence, DAPI, and bright-field images were merged. Samples were imaged by laser scanning confocal microscopy (LEICA TCS SP8, Germany). Bar =25 μm

To determine the intracellular localization of the four Md14–3-3 proteins, Md14–3-3 s::GFP fusion construct were transiently expressed in *Nicotiana benthamiana* leaves. The fluorescent signals were observed in the cytoplasm and nucleus (Fig. 8), consistent with previous studies [11].

## Discussion

Plants need a series of regulatory factors to sense and respond to complex environmental changes, which seems to be relevant to the abundant existence of 14–3-3 family proteins. Almost all eukaryotes have multiple 14–3-3 isoforms, thus increasing the functional complexity of this regulatory protein family. The family has 15 and 8 14–3-3 genes in the *Arabidopsis* and rice genomes, respectively [3, 5, 49]. In this study, we identified 18 Md14–3-3 genes in apple. The lengths of isoforms vary from 252 to 302 amino acids (Table 1). In plants, the 14–3-3 proteins form homo- and hetero-dimeric proteins. Each monomer in the dimer contains a conserved core region composed of nine antiparallel α-helices (α1–α9) forming an amphiphilic groove. It is capable of binding a separate phosphorylated target protein [5, 6], which is the premise for the 14–3-3 proteins to participate in diverse pathways. The primary diversity among Md14–3-3 isoforms occurs at the N and C termini (Additional file 2: Figure S2), which are related to dimerization and target binding, respectively [50, 51]. As a result, these slight differences in the internal loops and the highly distinct termini are thought to contribute to the 14–3-3 isoform specificity by regulating differential affinity between individual 14–3-3 isoforms towards their possible targets [5, 46, 52]. Recent studies in *Arabidopsis* suggested 14–3-3 target specificity and further confirmed that the extreme C termini of 14–3-3 proteins play a crucial part in ligand interaction [53–55], although the exact mechanism is not entirely clear.

Phylogenetic analysis showed that Md14–3-3 s family members could be classified into two different evolutionary clades (group ɛ and non-ɛ) (Fig. 1; Fig. 2), consistent with the identified 14–3-3 family in other species [5, 41, 42, 44]. The exon-intron structural divergence analysis provided an insight into the evolutionary relationships of the Md14–3-3 genes. The gene structure appeared to be generally conserved among genes belonging to the same clade. As show in Fig. 1, The intron-exon structure of the ɛ members is different from that of the non-ɛ group. For example, all 10 Md14–3-3 s in ɛ group contained six exons, and most members within non-ɛ group had four exons, except for *MdGF14c* (Fig. 1). Additionally, most Md14–3-3 gene members in each group exhibited nearly identical exon lengths. The intron lengths and arrangements are different between subfamilies. Similar results were also be found in other species [42, 44]. These results suggested that exon-intron structure could reveal the evolutionary divergence of the Md14–3-3 gene family. Tandem, segmental duplication, and whole genome duplications events have made important contributions to the expansion of gene family members in plant evolution [45]. Our results further elucidated the expansion mechanism of the 14–3-3 family in apple. Two pairs of tandemly duplicated Md14–3-3 genes and three pairs of segmentally duplicated Md14–3-3 genes were detected (Fig. 3). Additionally, the number of chromosomes in apple genome was affected by recent whole-genome duplication events, resulting in an increase from 9 to 17 chromosomes [56]. These results indicated that gene duplications have played a crucial role in the expansion of the Md14–3-3 genes. Taken together, the evolution and phylogeny of the 14–3-3 family exhibit diversity and complexity, reflecting their functional divergence.

The 14–3-3 proteins are associated with several different proteins in signal transduction pathways [57]. Schoonheim et al. [18] identified a large number of target proteins of 14–3-3 isoforms via yeast two-hybrid screens. A number of proteins have well-defined functions in plant hormone signaling pathways, such as the auxin transport proteins PIN1 [58] and NPH3 [59], the major BR signaling related proteins [60], and the ABF transcription factor family members [61]. Interestingly, we found numerous hormone-related elements in the promoters of the Md14–3-3 family genes (Additional file 4: Table S2), which suggested that plant 14–3-3 proteins play a crucial regulatory role in many hormone-related signaling pathways. This conclusion was confirmed through transcriptome data and qRT-PCR analysis in our study (Fig. 4, Fig. 6). Additionally, 14–3-3 proteins have been shown to participate in the perception of light signaling through interacting with light-related proteins, such as PIF and CONSTITUTIVE PHOTOMORPHOGENIC1 (COP1) [62]. These results were also supported by the presence of multiple light-responsive and circadian-responsive elements in the promoter regions of the 14–3-3 genes (Additional file 4: Table S2).

A recent study summarized the regulation mechanisms of 14–3-3 proteins during the development of multiple organs in a number of plant species, including *Arabidopsis*, rice, soybean, rapeseed, and castor. Multiple 14–3-3 isoforms are expressed and function across the seed, flower, leaf, and root [57]. Our data demonstrated that most Md14–3-3 s were expressed in all detected tissues and were particularly mainly expressed in stems and flowers (Fig. 5), suggesting that the expression of 14–3-3 s is essential and important to maintain or respond to plant growth requirements. Notably, several Md14–3-3 isoforms with close evolutionary relationships (e.g. *MdGF14a* and *MdGF14i*) showed similar expression patterns in different tissues, while other Md14–3-3 s showed tissue-specific transcript accumulation patterns. For example, *MdGF14e* and *MdGF14k* displayed higher expression levels in flowers, which suggested that they might play a role in flower development. *MdGF14m* was specifically highly expressed in stems, suggesting its involvement in stem growth. Furthermore, *MdGF14d* and *MdGF14j* in the segmental duplication group, were mainly expressed in stems and flowers, signifying their putative role in the regulation of stem and flower development. Taken together, these results suggested functional diversity (overlapping or specific functions) of Md14–3-3 s proteins during apple growth and development, which was also supported by the sequence conservation and diversity of 14–3-3 isoforms in many species [41, 42, 44].

Apple flower induction is mediated by a huge gene network that receives signals from multiple pathways to determine the fate of bud in the second year. Phytohormones and sugars participate in growth and floral transition in apple [63]. Our previous study showed that 6-BA treatment increased the proportion of short branches and promoted floral transition [47]. Sugar, as an energy substance, is involved in flowering regulation [64]. Several studies showed that 14–3-3 genes were directly involved in floral development. In *Arabidopsis*, 14–3-3*ν* and *μ* knockout lines displayed late flowering [22]. In tomato, 14–3-3 genes are able to compensate for the effect of knocking out SELF-PRUNING (SP), which is a homologous gene of TFL1, by inducing the indeterminate growth of inflorescence [23]. In rice, functional analysis of GF14c (a 14–3-3 protein) indicated that plants overexpressing GF14c cause delayed flowering, while the knockout mutants induce early flowering compared with the wild-type control [11]. Therefore, it would be interesting to understand the role of Md14–3-3 s in the transition from vegetative to reproductive growth in apple. In this study, RNA-based sequencing data generated from 6-BA and sugar response, combined with qRT-PCR results using GA_3_ treatment, enabled us to identify Md14–3-3 genes that respond to hormones or sugar. The results showed that the expression trends of 14–3-3 s are diverse and vary depending on developmental stages under treatment with sugar and hormones (Fig. 5; Fig. 6). 6-BA and GA have antagonistic actions on apple flowering, and act as a positive promoter and a negative regulatory factor, respectively [40, 47]. This antagonistic effect is also consistent with the expression patterns of most Md14–3-3 genes. For example, at 30 DAFB (a key point for floral induction), the expression levels of *MdGF14a* and *MdGF14i* were significantly increased by GA_3_ treatment (Fig. 6). However, under 6-BA treatment, *MdGF14a* and *MdGF14i* showed the opposite expressions patterns (Fig. 5). Moreover, several Md14–3-3 genes (e.g. *MdGF14b*, *MdGF14c*, *MdGF14d*, *MdGF14e*, and *MdGF14j*) were upregulated at the early stage after 6-BA treatment, and downregulated after GA_3_ treatment (Fig. 5, Fig. 6). Collectively, we preliminary hypothesized that these Md14–3-3 genes might have significant roles in the regulation of floral transition, and their functions merit further investigation.

In apple, MdFT is a paralog of MdTFL1, but has a converse function in flower development [65]. Overexpression of the MdFT-encoding gene in apple resulted in precocious flowering. Recent research showed that *MdFT1* transcripts are expressed to appreciable levels in the apical bud, but are not significantly affected by 6-BA treatment [66] and GA treatment [67] during floral transition. Interestingly, the transcription level of *MdTFL1* was decreased by 6-BA [66], while it was apparently increased by GA treatment (or fruit load) during flower induction [67, 68]. In other words, GA inhibition of apple flowering appears to be mediated by inducing a significant increase in *MdTFL1* levels. In plants, 14–3-3 proteins act as interactor of both TFL1 and FT [24, 36]. In our study, the identification of Md14–3-3 s (MdGF14a, MdGF14d, MdGF14i, and MdGF14j) as MdTFL1 and MdFT binding partners was confirmed using yeast two-hybrid assays and BiFC assays (Fig. 7). These results indicated that 14–3-3 proteins are involved in flowering regulation through direct association with floral genes. The subcellular distribution of the four Md14–3-3 isoforms showed cytoplasmic and nuclear localizations (Fig. 8). Several pieces of evidence indicated that the subcellular distribution of 14–3-3 proteins seems to be highly dependent on the interaction with their targets [8, 11, 12, 19]. Previous reports also found that the binding of 14–3-3 proteins with partner proteins can change their subcellular localization [69]. To understand whether the protein interactions would affect the subcellular localization of MdTFL1 and MdFT, a BiFC experiment was performed. Notably, the MdTFL1-Md14–3-3 s or MdFT-Md14–3-3 s BiFC signals were mainly detected in the cytoplasm and weakly in the nucleus (Fig. 7b, c, d), which was consistent with previous reports [11, 36]. These results suggested that MdTFL1/MdFT and Md14–3-3 s interactions increase the cytoplasmic retention of MdTFL1 or MdFT and inhibit their shuttling from the cytoplasm into the nucleus.

**Fig. 9:**
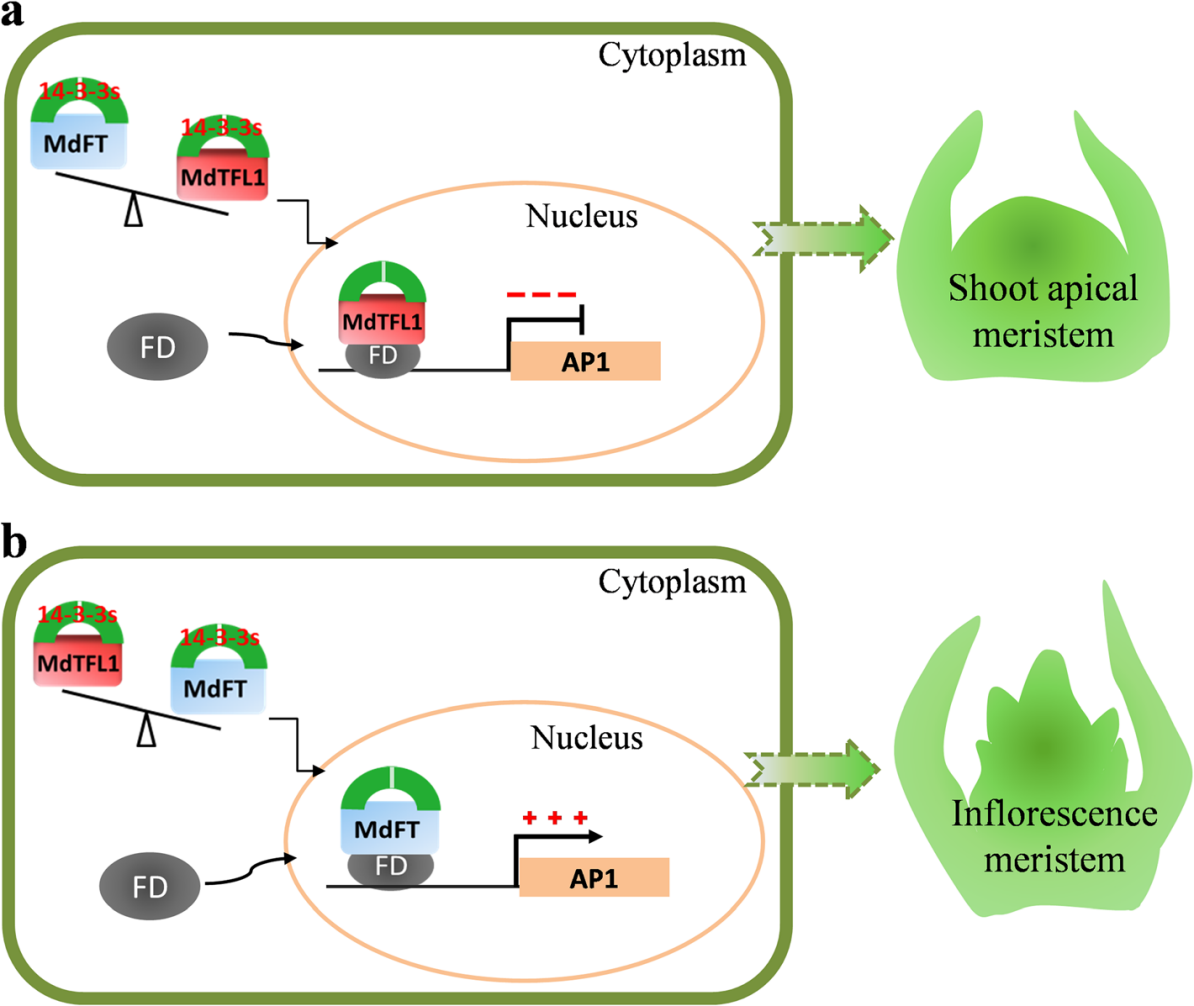
A model for interaction of 14–3-3 with TFL1 and FT. The interaction complexes comprising TFL1–14–3-3 s or FT-14-3-3 s mainly occur in the cytoplasm and form a larger ternary complex with FD in the nucleus, thereby regulating the expression of the downstream floral meristem identity gene *AP1* for flower transition [36]. TFL1 competes with FT for 14–3-3 binding. TFL1–14–3-3-FD complex acts as a floral repressor (**a**), While FT-14-3-3-FD complex acts as a floral activator (**b**). The balance between TFL1 and FT controls the vegetative and reproductive growth in the apical meristem, modulating the plant architecture and optimizing of crop productivity

Much is known about the antagonistic functions of TFL1 and FT in the regulation of flowering time. Therefore, interactions of MdTFL1/MdFT with Md14–3-3 s led to the hypothesis that MdTFL1 antagonizes MdFT through competition with Md14–3-3 binding. Current and previous studies have provided several pieces of evidence that strongly support this hypothesis. For example, in *Arabidopsis*, TFL1 and FT act as transcription repressors or transcription activators, through interactions with FD and 14–3-3 proteins, respectively, to regulate the expression of floral meristem identity genes *LFY* and *AP1* [70]. In tomato, SP (a TFL1 homolog) and SFT (an FT homolog) directly interact with 14–3-3 isoforms [71]. In rice, there exist associations between RCN (a rice TFL1 homolog) or Hd3a (a rice FT homolog) and 14–3-3 proteins, and the antagonism between RCN and Hd3a is dependent on 14–3-3 binding [36]. These results suggested that TFL1 antagonizes FT for 14–3-3 binding. It is thought that the balance between FT and TFL1 in the regulation of phase transition is systemic and widespread in plants [35]. A high FT/TFL1 ratio in the meristem promotes determinate growth and induces transition to the reproductive phase, eventually converting the SAM into a terminal flower, while a low FT/TFL1 balance maintains indeterminate plant growth. The resulting balance serves an important role in accurate modulating of a plant’s response to flower-induced signals (Fig. 9). However, how MdTFL1 antagonizes MdFT for 14–3-3 binding needs further research in apple.

## Conclusion

This study presents a comprehensive classification of the Md14–3-3 gene family in apple and provides evidence for their possible roles in apple flowering. There are 18 Md14–3-3 genes in the apple genome, which could be grouped into ε and non-ε groups. The diverse expression patterns of Md14–3-3 s in various tissues and in response to treatment with sugar and hormones suggested that 14–3-3 family members serve as positive or negative regulators mediating floral transition. Further clues indicated that MdTFL1 (MdTFL1–1 and MdTFL1–2) and MdFT, which are important floral integrators, act as 14–3-3 s binding partners. Nevertheless, further studies on the functional mechanism of apple 14–3-3 proteins during floral transition should be performed.

## Methods

### Identification and chromosomal location of 14–3-3 family in apple

To identify potential 14–3-3 s gene family members in the apple genome, we retrieved 15 previously published *Arabidopsis* 14–3-3 protein sequences from the *Arabidopsis* genome database (http://www.arabidopsis.org/), which were used as queries in BLASTp (E-value <1e-5) searches against the apple genome (GDDH13V1.1; https://www.rosaceae.org/). Furthermore, candidate 14–3-3 family members were confirmed for their highly conserved domain using Pfam (http://pfam.xfam.org/) and the Conserved Domain Database (CDD, https://www.ncbi.nlm.nih.gov/Structure/cdd/wrpsb.cgi). Only those sequences having a full-length 14–3-3 domain were selected as Md14–3-3 proteins and used for the subsequent analyses. The candidate 14–3-3 gens annotations and their chromosomal locations were obtained from the apple genome.

### Sequence alignment, gene structure, *cis*-element analysis, and phylogenetic tree construction

Multiple alignments of Md14–3-3 proteins sequences were performed in DNAMAN software (V 6.0). The Md14–3-3 exon-intron structures were generated using the online tool of Gene Structure Display Server (http://gsds.cbi.pku.edu.cn) [72]. The 2000 bp intergenic regions upstream of the start codon of the Md14–3-3 genes were derived from the apple genome. The PlantCARE online program (http://bioinformatics.psb.ugent.be/webtools/plantcare/html/) was used to search for assumed *cis*-elements in promoter region. Sequence alignment of 14–3-3 proteins was created using the Muscle tool in MEGA 7.0. The phylogenetic tree was constructed based on the alignment results using the MEGA 7.0 program by the maximum likelihood method with Poisson model and partial deletion.

### Tandem duplication and synteny analysis

Tandem duplication and synteny relationships were analyzed using Circos 0.63 (http://circos.ca/) [73]. According to previous published criteria [74], gene duplication events were defined based on their chromosomal locations: genes located on the same chromosome were considered tandem-duplicated genes, and genes located on different chromosomes were called segmental duplicated genes. The Plant Genome Duplication Database (http://chibba.agtec.uga.edu/duplication/) was used to performed synteny analysis between apple and *Arabidopsis*.

### Expression profiles of Md14–3-3 genes in RNA-seq datasets

The expression profiles of putative Md14–3-3 genes was determined using RNA-seq datasets, including transcriptional response to 6-BA, glucose, and sucrose treatments during key periods of floral transition. RNA-seq data for the expression profiles in response to 6-BA was retrieved from the NCBI Short Read Archive (SRA) under the accession number SRR6510620 (https://www.ncbi.nlm.nih.gov/sra/?term=SRR6510620) [47]. RNA-seq data in response to glucose treatment was retrieved from the NCBI Sequence Read Archive (SRP226830) (https://www.ncbi.nlm.nih.gov/sra/?term=SRP226830). Six-year-old ‘Nagafu No. 2’ trees were used for glucose treatment. Glucose at 15,000 and 30,000 mg L^− 1^ was sprayed onto the trees at 25 and 30 DAFB, respectively. Control plants were also treated with an equivalent amount of water. After two glucose treatments, the terminal buds of the short shoots (< 5 cm) were collected for further RNA-seq analysis. In addition, RNA-seq datasets under sucrose treatment (data not shared online) are available to analyze the expression profiles of Md14–3-3 genes. The specific operations were as follows, sucrose treatment liquid (15,000 mg L^− 1^ and 20,000 mg L^− 1^ sucrose for 29 and 36 DAFB, respectively) were sprayed on apple leaves using a handheld sprayer until run-off. At the same time, water was sprayed as a blank control. Samples of the short shoot apices were collected at 30, 50, and 70 DAFB for further RNA-seq analysis. The first bud sampling stage at 30 DAFB was sucrose treated only once, while plants at the other sampling dates were treated twice. Three biological replicates were performed for each treatment. Library construction and sequencing were performed using the Illumina HiSeq platform. The analysis of RNA-seq data was based on a previously published method [47]. The FPKM values were used to estimate the gene expression level. FPKM values for all types of treatment involved three sampling time points: 30, 50, and 70 DAFB. The heatmap of the expression of Md14–3-3 genes was constructed using Heml 1.0 software.

### Plant growth conditions and GA_3_ treatment

Plant samples were collected from six-year-old ‘Nagafu No.2’/‘M26’/*M. robusta* Rehd trees, which grows in the Yangling Modern Agriculture Technology Apple Park (Shaanxi Province, China).

The six tissue samples, including leaves, stems, leaf buds, flower buds, flowers, and fruit, were collected for organ-specific expression analysis. Mature leaves were collected from the adjacent terminal buds of the spur. Stems were collected from fresh shoots pulled out in the spring. Additionally, leaf buds were collected from bourse shoot apices with adjacent developing fruit, which find it difficult to form flowers in the next year. Flower buds were collected from plump terminal buds of the short shoot. Moreover, young fruit was also collected at 40 DAFB. All samples were frozen at − 80 °C for qRT-PCR analysis.

For GA_3_ treatment, 500 mg L^− 1^ GA_3_ was sprayed at 25 and 30 DAFB on a clear morning. At the same time, water was sprayed as a control. GA_3_ and control solutions were applied using a handheld sprayer until run-off. About 2 h after the second treatment (30 DAFB), the first samples of spur terminal buds were collected on the same day (30 DAFB), and then at 50, and 70 DAFB. Samples were stored at − 80 °C for further qRT-PCR analysis.

### RNA extraction, cDNA synthesis, and gene expression analysis using qRT-PCR

The expression levels of Md14–3-3 genes in different tissues and different developmental stages of flowering induction were analyzed by qRT-PCR. We extracted total RNA using a polysaccharide polyphenol plant total RNA Extraction Kit (Foregene, Chengdu, China) following the manufacturer’s instructions. First-strand cDNA was generated using a PrimeScript RT Reagent kit (Takara, Japan). The qPCR step was completed using a CFX Connect Real-Time System machine (Bio-Rad, USA). The 20 μL qRT-PCR reactions mixture composed of 10 μL of TB Green Premix Ex Taq II (Takara), 2 μL of cDNA (diluted 1:8), 2 μL of gene-specific primers (10 μM), and 6 μL of water. The housekeeping histone H3-encoding gene (LOC103406086, XM_008345103) was used as a reference gene to calculate the relative expression of the selected genes [75]. For each time point, three independent biological repeats with three technical repeats were performed. Relative gene expression levels were calculated using the 2^−ΔΔCt^ method [76]. Student’s *t*-test was used to estimate the statistical significance of relative expression between control and treatment at each time point. Specific primer pairs were designed using Primer-BLAST online tool at the NCBI database (https://www.ncbi.nlm.nih.gov/tools/primer-blast/). Graphpad Prism 7.0 was used to generate figures. The primers are listed in Additional file 6: Table S4.

### Yeast two-hybrid assay

To confirm the interaction between MdTFL1/MdFT and Md14–3-3 proteins, the coding sequences of MdTFL1–1, MdTFL1–2, and MdFT were cloned into the bait vector pGBKT7, and Md14–3-3 s (MdGF14a, MdGF14d, MdGF14i and MdGF14j) sequences were cloned into the prey vector pGADT7. Yeast strain Y2H gold with the recombinant bait vector was used for a self-activation and self-toxicity check. Next, the bait-prey interactions were tested on SD medium without Leu, Trp, His, and Ade, according to the Matchmaker™ Gold Yeast Two-Hybrid System (Clontech). The primers used in this assay are listed in Additional file 6: Table S4.

### Bimolecular fluorescence complementation

The full-length coding sequences of MdTFL1/MdFT and candidate Md14–3-3 interacting proteins were cloned into the pSPYNE and pSPYCE vectors, respectively, for protein-protein interaction assays [77]. The resulting plasmids were transformed into *Agrobacterium tumefaciens* (strain GV3101), incubated and harvested in infiltration mixture (10 mM MES, 10 mM MgCl_2_ and 0.2 mM acetosyringone, pH 5.6). Then, the two candidate *Agrobacterium* cultures were mixed in equal volumes and co-transformed into *Nicotiana benthamiana* leaves. Infected tissues were analyzed at 48 h after infiltration. Yellow fluorescent protein (YFP) signals were observed using laser scanning confocal microscopy (LEICA TCS SP8, Germany) with PMT spectrometry detector. The object lens (20×) was used for microscopic observations. The YFP and auto-fluorescence of chlorophylls was excited at 514 nm and the spectral detector was set at 530–560 nm and 660–700 nm, respectively. Images were acquired using Leica Application Suite (LAS) software (Version 3.3). The primers used for BiFC are listed in Additional file 6: Table S4.

### Subcellular location

The full-length Md14–3-3 coding sequences, without the stop codon, were fused with GFP at the 3′-terminus and cloned into pCAMBIA2300 vector. *Agrobacterium* (GV3101) transformed with the target vectors were suspended in infiltration buffer to final concentrations of A600 = 0.6. Next, the injection of *Nicotiana benthamiana* leaves was performed. The infected plants were cultured at 25 °C for 48 h before observation using laser scanning confocal microscopy (LEICA TCS SP8, Germany). The 488 nm laser line was used to excite GFP. Other parameters are the same as the above BiFC assay. Relevant primer information is listed in Additional file 6: Table S4.

## Supplementary Information

**Additional file 1: Figure S1.** Chromosome map of Md14-3-3 genes in apple.

**Additional file 2: Figure S2.** Sequence alignment of Md14-3-3 proteins in apple. Identical residues are shown in blue and similar residues are in red. Nine antiparallel α-helices were marked as α1-α9.

**Additional file 3: Table S1.** The gene name and gene ID of 14-3-3s in apple and other plant species.

**Additional file 4: Table S2.** Analysis of the *cis*-elements in the Md14-3-3 promoter. The 2,000bp upstream from the start codon of Md14-3-3 genes were analyzed using the PlantCARE database.

**Additional file 5: Table S3.** FPKM values of Md14-3-3s from RNA-seq statistics during floral transition in 6-BA, glucose and sucrose treatment.

**Additional file 6: Table S4.** Primers used in the present study.

**Additional file 7: Figure S3.** Yeast two-hybrid and BiFC assays of interactions between MdTFL1-2 and candidate Md14-3-3 proteins. (**a**) Yeast two-hybrid assays. MdTFL1–2 were fused to the pGBKT7 vector. The empty pGADT7 vector was used as control. -LT, yeast medium lacking leucine and tryptophan. -LTAH, yeast medium lacking leucine, tryptophan, adenine and histidine. (**b**) BiFC assays. The coding regions of MdTFL1–2 were cloned into pSPYNE, and MdGF14a, MdGF14d, MdGF14i, and MdGF14j were cloned into the pSPYCE vector. The empty pSPYCE and pSPYNE vector served as the control. The YFP fluorescence, chlorophyll autofluorescence (CHl), and bright-field images were merged. Bar = 25 μm.

## Data Availability

All relevant data analyzed during this study are included in this article and in Additional files. RNA-seq data in response to 6-BA and glucose treatment were acquired from NCBI Short Read Archive (SRA) (the accession number: SRR6510620 and SRP226830, respectively) (https://www.ncbi.nlm.nih.gov/sra/?term=SRR6510620 and https://www.ncbi.nlm.nih.gov/sra/?term=SRP226830). The apple RNA-seq data for sucrose treatment used during the current study are available from the corresponding author on reasonable request.

## Abbreviations

GA: Gibberellin
ABA: Abscisic acid
BR: Brassinosteroids
6-BA: 6-benzylaminopurine
DAFB: Days after full bloom
FPKM: The fragments per kilobase of transcript sequence per million base pairs sequenced
TFL1: TERMINAL FLOWER1
FT: FLOWERING LOCUS T
CO: CONSTANS
PEBP: Phosphatidylethanolamine binding protein
SAM: Shoot apical meristem
PIF: PHYTOCHROME-INTERACTING FACTOR
AP1: APETALA1
COP1: CONSTITUTIVE PHOTOMORPHOGENIC1
SP: SELF-PRUNING
qRT-PCR: quantitative reverse-transcription PCR
GFP: Green fluorescent protein
BiFC: Bimolecular fluorescence complementation
