# Supplementary figures and images for: Genome-wide identification of the 14–3-3 gene family and its participation in floral transition by interacting with TFL1/FT in apple
Source: BMC Genomics. 2021 Jan 8;22:41. doi: 10.1186/s12864-020-07330-2 (PMC7796649; doi:10.1186/s12864-020-07330-2)

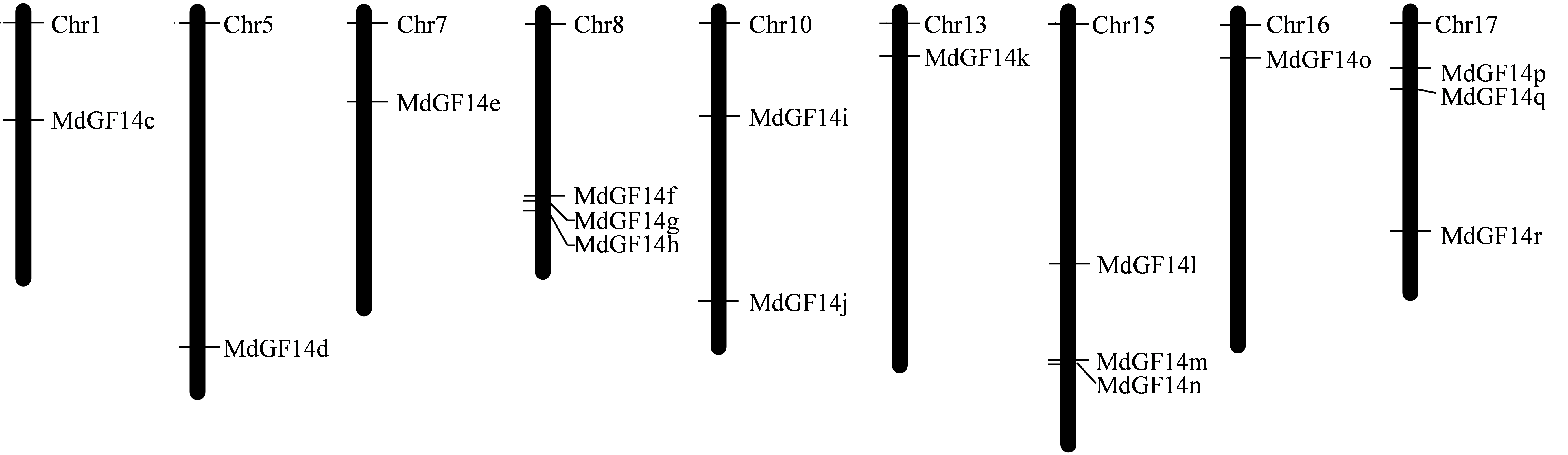

Supplement: Supplementary file 1 — Additional file 1: Figure S1. Chromosome map of Md14-3-3 genes in apple. [file 12864_2020_7330_MOESM1_ESM.tif]

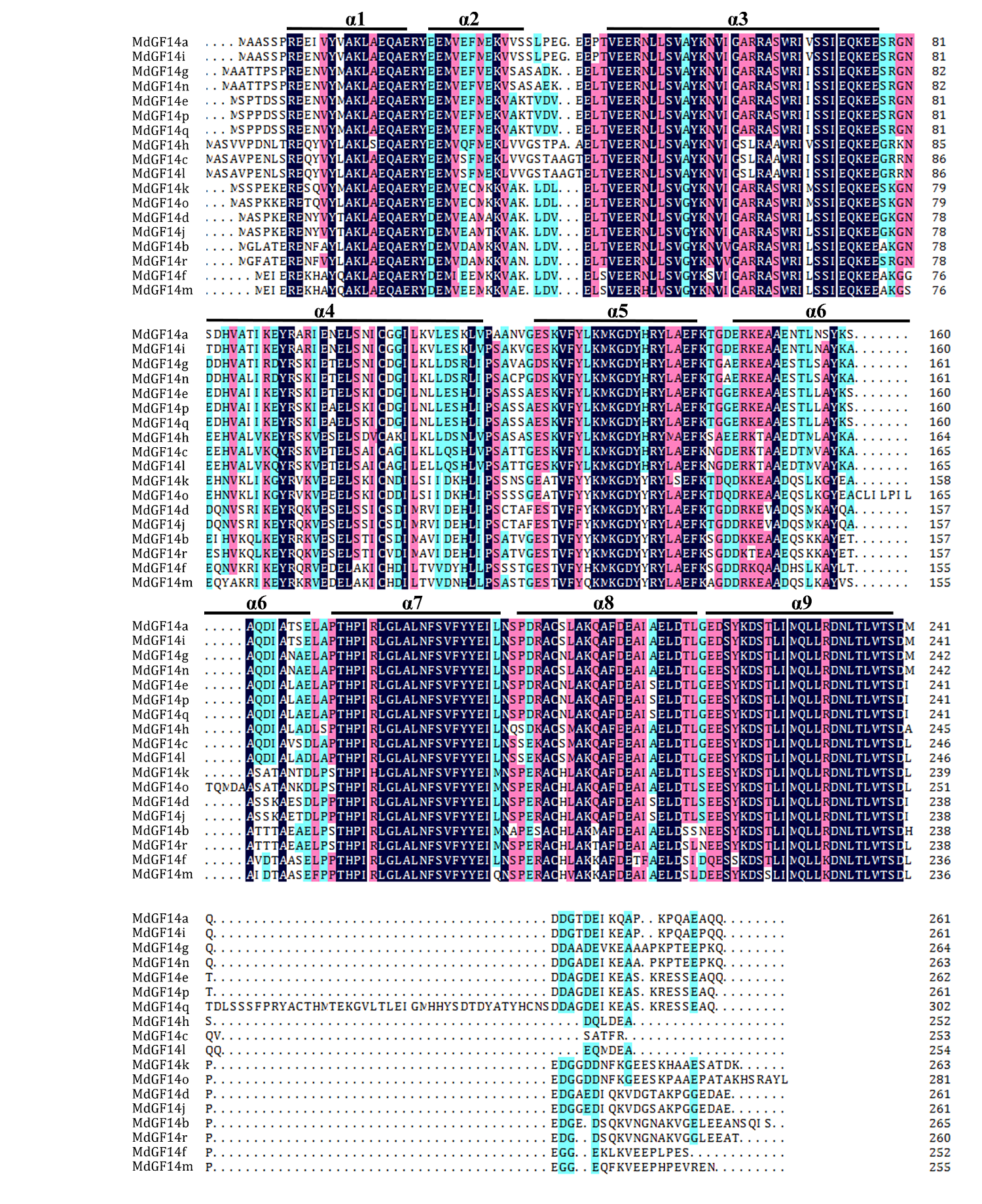

Supplement: Supplementary file 2 — Additional file 2: Figure S2. Sequence alignment of Md14-3-3 proteins in apple. Identical residues are shown in blue and similar residues are in red. Nine antiparallel α-helices were marked as α1-α9. [file 12864_2020_7330_MOESM2_ESM.tif]

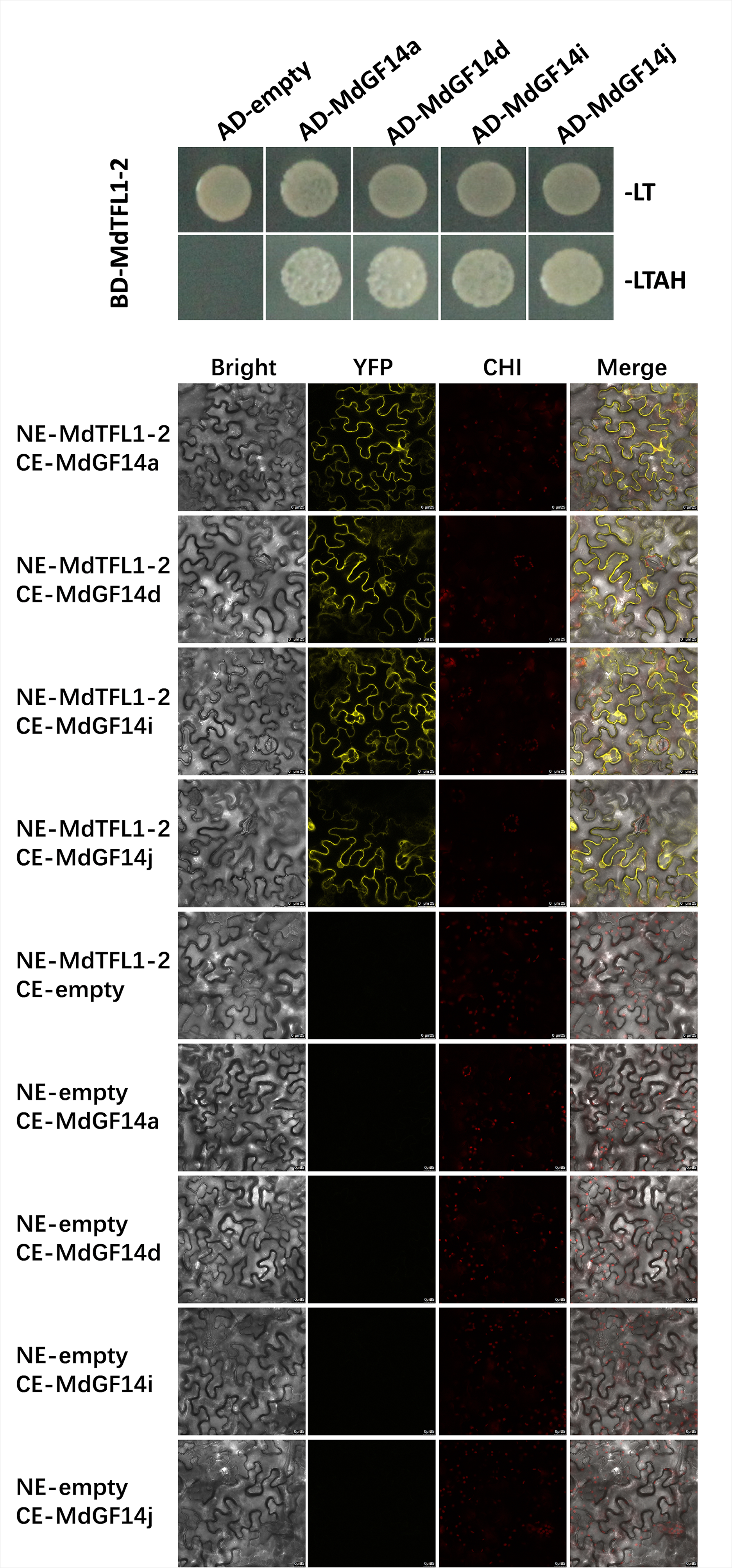

Supplement: Supplementary file 7 — Additional file 7: Figure S3. Yeast two-hybrid and BiFC assays of interactions between MdTFL1-2 and candidate Md14-3-3 proteins. (a) Yeast two-hybrid assays. MdTFL1–2 were fused to the pGBKT7 vector. The empty pGADT7 vector was used as control. -LT, yeast medium lacking leucine and tryptophan. -LTAH, yeast medium lacking leucine, tryptophan, adenine and histidine. (b) BiFC assays. The coding regions of MdTFL1–2 were cloned into pSPYNE, and MdGF14a, MdGF14d, MdGF14i, and MdGF14j were cloned into the pSPYCE vector. The empty pSPYCE and pSPYNE vector served as the control. The YFP fluorescence, chlorophyll autofluorescence (CHl), and bright-field images were merged. Bar = 25 μm. [file 12864_2020_7330_MOESM7_ESM.tif]
